# Fence the Family

**Published:** 1871-07

**Authors:** 


HALL'S

JOURNAL OF HEALTH.

VOL. XVIII. — JULY, 1871. — NO. VII.

### FENCE THE FAMILY.

It is an expedient in some sickly portions of the South and West, where troublesome and mortal fevers prevail, to cultivate a little forest fence of sunflowers all around the dwelling, a short distance from it, to shut out from the family those bad airs — miasms — which generate so much sickness and disease; not that there is any specific virtue in the sunflower, for any other thick growth or vigorous vegetable hedge, would be equally efficient; the philosophy of it being, that living vegetation absorbs the miasm, and thus prevents its transmission to the family habitation.

The same care should be exercised in shutting out from the family the causes of moral disease which insinuate themselves into the mind and heart, the affections and the emotions, where they burrow and feed and fester and corrupt, and eventually destroy the life of body and soul together.

Many years ago we gave a little Sunday-school book to a patient of ours, for her little children to read. On a subsequent visit, we asked how the little ones were pleased with it, and she replied apologetically, that she had not yet given it to them, as she had made it a rule never to put any book into the hands of her children until she had first read it herself, to see whether there was anything in it objectionable in her opinion. She was a Roman Catholic, the wife of one of the leading business men. She died not long after, and our memories of the wife of Banker Benoist, more than a quarter of a century ago, bring up one of the most perfect examples of a Christian mother ever met with by us.

Looking into a New York weekly pictorial to-day, which visits a hundred thousand families fifty-two times every year, a continued story is published, so obscene, so corrupting, so insufferably suggestive of nastiness, that even the secular press appeals to the publishers, in behalf of its multitudes of intelligent readers, to purge themselves of such an outrage against public decency, by dropping the tale from its columns instantly. In another pictorial from Boston, a poem, or piece of machine-poetry is illustrated in all the beauty of art and tinted paper, closing thus, —

> "And I think that saving a little child,
> And bringing him to his own,
> Is a derned sight better business
> Than loafing around The Throne."

Thus to travesty religious sentiment must grate upon every good man's feelings, and make him stand aghast with fear.

And in speaking of a reckless, drunken, profane swearer who had died in the effort to save others, the expression of a sentiment is given thus : —

> "And Christ ain't a going to be too hard
> On a man that died for men."

To describe in slang phrases the dearest and most sacred of all Christian sentiments and subjects, may well make us feel that we have fallen on evil times.

Then again, there is a Boston periodical written for by the most cultivated minds in the nation, uniformly commended by very many religious newspapers, and yet, every now and then, there are stabs at the Christian religion, which operate like poisoned arrows, and unsettle the minds of the young and weak in reference to some of the most fundamental truths in the Christian system.

Not a week passes, wherein a careful reader of the New York daily papers will not find in some of the most unexceptionable of them very much that is calculated to excite prejudice and ridicule as to the Christian system, with incessant tilts against the sanctity of the Sabbath, against the ministers of religion; with inculcations tending to tear away all the barriers around the family relation. Even medical publications are not always free from irreligious poisonings. Scientific men come in for a share of blame in this respect; all ought to be watched against. Too many of the religious press, in apparent mortal fear of offending publishers who advertise with them and send many valuable books for "notice," are too much inclined to commend what is commendable, and gingerly pass over, or do not notice at all, sentiments inculcated, which, if carried out legitimately, would destroy the Christian system altogether.

"THE DESCENT OF MAN"

Has its gold and its alloy; its food and its poison; its truth and its error; and on the whole, is as mischievous, as infidel, as Darwin's "Botanical Garden" of many years ago.

The Bible teaches that man was made perfect, and degenerated into moral corruption and physical deterioration. Now Darwin says that he was made next thing to an animal idiot, and by successive evolutions has turned "up" to what he is, to rise higher and higher as the ages pass on.

The Bible's man was made in the image of his Maker; Mr. Darwin's in that of the monkey, or even lower down.

The statement of the Bible is, that man "fell" from his paradisiacal state; Darwin asserts that he is rising towards it.

The Bible teaches that the cruelties and crimes and corruptions of man are the legitimate results of his departures from Him, of his alienation from his Maker and Father, and that the tendencies are downward forever; Mr. Darwin asserts that all these are the results of imperfection, of original defect or immaturity, and that by his own civilization man has been improving from the first day of creation.

The Bible provides a Saviour from all sin; Mr. Darwin's theory is that man is his own Saviour, and in the very nature of things evolves from a mollusk to an immortal, from an oyster to an angel. To every parent, to every educated parent, to every Christian parent, especially to Christian mothers, we appeal in the name of a common humanity, in the name of a true civilization, in the name of our holy religion,

"FENCE THE FAMILY."

See to it that safe books are placed in your libraries, that the magazines, the weekly and the daily newspapers, which are placed on your centre-tables, and the illustrated publications which are to impress your children earliest, shall illustrate — not the vice and crime, the murder and the bloodshed, the nakedness and obscenities of the times, but the beauties of art and of nature; of the gallery and the garden; of the family and the church. And for a beginning, and to make it at once practical and possible, seeing that illustrated publications are the fashion of the times, take for your illustrated paper, over which your little ones will delight their eyes, and be led to inform their minds by seeking to read the explanations,

"THE CHRISTIAN WEEKLY,"

Only two dollars a year, eight pages; four of them illustrated in a style of perfection not excelled in that line anywhere in this country; beautiful, smooth white paper, clear large type; filled with truthful histories, with reliable information in morals, news, and general literature; with narratives for the little ones, solid truth for the mature, and comfort and consolation for the old, the feeble, the invalid, and for all.

For smaller children, "The Child's Paper," published monthly, beautifully illustrated, issuing three hundred and fifty thousand copies twelve times a year, only twenty-five cents a year. One dollar will send a copy to eight families twelve times a year. How much delight a single dollar can give!

Address R. C. Loesch, 150 Nassau St., New York.

These are papers which will help to keep your little ones from the street, the circus, the low theatre, the grog-shop, and the gallows.

